# Chronic kidney disease in US adults with type 2 diabetes: an updated national estimate of prevalence based on Kidney Disease: Improving Global Outcomes (KDIGO) staging

**DOI:** 10.1186/1756-0500-7-415

**Published:** 2014-07-02

**Authors:** Robert A Bailey, Yiting Wang, Vivienne Zhu, Marcia FT Rupnow

**Affiliations:** 1 Janssen Scientific Affairs, LLC, 1000 Route 202 South, Raritan, NJ 08869, USA; 2 Janssen Research & Development, LLC, Titusville, NJ, USA

**Keywords:** Diabetes, Type 2 diabetes mellitus, Chronic kidney disease, Prevalence, Estimated glomerular filtration rate, Albuminuria

## Abstract

**Background:**

Kidney Disease Improving Global Outcomes (KDIGO) 2013 updated the classification and risk stratification of chronic kidney disease (CKD) to include both the level of renal function and urinary albumin excretion (UAE). The update subclassifies the previous category of moderate renal impairment. There is currently limited information on the prevalence of CKD based on this new classification in United States (US) adults with type 2 diabetes mellitus (T2DM). The objective of this study was to provide such estimates, for T2DM both overall and in those ≥ 65 years of age. We used the continuous National Health and Nutrition Examination Survey (NHANES) 1999–2012 to identify participants with T2DM. Estimated glomerular filtration rate (eGFR) and UAE were calculated using laboratory results and data collected from the surveys, and categorized based on the KDIGO classification. Projections for the US T2DM population were based on NHANES sampling weights.

**Results:**

A total of 2915 adults diagnosed with T2DM were identified from NHANES, with 1466 being age ≥ 65 years. Prevalence of CKD based on either eGFR or UAE was 43.5% in the T2DM population overall, and 61.0% in those age ≥ 65 years. The prevalence of mildly decreased renal function or worse (eGFR < 60/ml/min/1.73 m^2^) was 22.0% overall and 43.1% in those age ≥ 65 years. Prevalence of more severe renal impairment (eGFR < 45 ml/min/1.73 m^2^) was 9.0% overall and 18.6% in those age ≥ 65 years. The prevalence of elevated UAE (> 30 mg/g) was 32.2% overall and 39.1% in those age ≥ 65 years. The most common comorbidities were hypertension, retinopathy, coronary heart disease, myocardial infarction, and congestive heart failure.

**Conclusions:**

This study confirms the high prevalence of CKD in T2DM, impacting 43.5% of this population. Additionally, this study is among the first to report US prevalence estimates of CKD based on the new KDIGO CKD staging system.

## Background

Type 2 diabetes mellitus (T2DM) is the leading cause of chronic kidney disease (CKD) in the United States (US), with previous estimates suggesting close to 40% of patients with T2DM having evidence of CKD [1]. Concordant with the high prevalence of CKD in this population, diabetes was attributed as the cause of end-stage renal disease (ESRD) in 44.2% of incident dialysis patients in 2011 [2]. The high burden of CKD in T2DM and the associated adverse outcomes result in high health-care costs to both public and private payers [2,3].

Based on the significant impact of CKD, Kidney Disease Outcomes Quality Initiative (KDOQI) and Kidney Disease: Improving Global Outcomes (KDIGO) recommend a focus on early identification of CKD. This early identification may allow for treatment directed at CKD to slow or prevent progression, and treatment of associated complications and comorbidities such as cardiovascular disease [4,5].

KDOQI originally proposed a uniform CKD staging and classification system based on estimated glomerular filtration rate (eGFR) [5]. The original classification ranged from Stage 1 CKD (defined as normal kidney function with other markers for kidney damage) to Stage 5 CKD (defined as kidney failure). This staging system served to provide clinicians with stage-specific action plans for treatment of CKD and associated comorbidities and complications. Additionally, this staging system facilitated research and served as a framework for developing a public-health approach to CKD.

Soon after the original KDOQI CKD staging system was published, Go *et al.*[6] reported that although the risk for death, cardiovascular disease, and hospitalizations increased when eGFR declined below 60 ml/min/1.73 m^2^ (Stage 3 CKD), these risks substantially increased when eGFR declined below 45 ml/min/1.73 m^2^[6]. Based on this evidence, Go *et al.* advocated for subclassification of Stage 3 to reflect these findings. A recent meta-analysis provided further evidence to support the division of Stage 3 CKD into 3a and 3b based on the increased risk for progression to ESRD, and cardiovascular and all-cause mortality at the GFR threshold of < 45 ml/min/m^2^[7,8].

Based on this new evidence, developed since KDOQI, KDIGO recently updated the classification system for CKD [4]. This new classification system is based on the *c*ause of CKD, *g*FR category, and *a*lbuminuria category (CGA). The cause category is based on the presence or absence of systemic disease. The GFR categories acknowledge the need to subdivide Stage 3 CKD, classified as G3 under the new classification system, into G3a (eGFR 45–59) and G3b (eGFR 30–44). The albuminuria categories are based on the presence and severity of albuminuria. This new classification was developed to allow risk stratification based on progression of CKD and cardiovascular and all-cause mortality.

The prevalence of diabetes in the older adult population (age > 65) is greater than 20%. This older adult group also has a higher prevalence of comorbid conditions (such hypertension, cardiovascular disease, and CKD) compared to younger populations [2,9]. Based on these specific considerations in the older adult population, the American Diabetes Association (ADA) recommends a different approach in this population. These specific recommendations include potentially less stringent treatment targets and special consideration in the selection of antihyperglycemic agents.

To date, there are no US estimates of the prevalence of CKD in T2DM based on this new classification system, particularly for stages G3a and G3b. The objective of this study was to provide current national estimates of the prevalence of CKD in the overall T2DM population, and in the subpopulation aged ≥ 65 years using this new KDIGO classification.

## Methods

National Health and Nutrition Examination Survey (NHANES) 1999–2012 data were used for this study. NHANES is a cross-sectional survey designed to monitor the health and nutritional status of the civilian non-institutionalized US population [10]. NHANES data include demographics, comorbidities, and medications that were reported by the survey participants during a home interview. Additionally, standardized physical examinations and laboratory tests were conducted in mobile examination centers. This study was exempt from the requirement of an Institutional Review Board since this research was based on data that were available from a publicly available source.

NHANES participants with T2DM were identified using previously published criteria to differentiate T2DM from type 1 diabetes mellitus (T1DM) [11,12]. The criteria used were: self-reported diagnosis of diabetes or “sugar diabetes” at age ≥ 30 years, not initiating insulin therapy within 1 year of diagnosis, and not pregnant (women) at the time of interview and examination. We focused on diagnosed diabetes with an aim to help health-care providers and population health managers to understand CKD and plan preventive management for their patients known to have T2DM; strategies for prediabetes and undiagnosed diabetes (such as by the criteria of hemoglobin A1c above 6.5% or single fasting plasma glucose measure above 180 mg/dl) are likely to be different. Since evidence suggests that the prevalence of reduced GFR, albuminuria, and micro/macrovascular complications differs between T1DM and T2DM [13], we made an effort to identify and exclude those with T1DM.

Comorbidities were determined based on participant self-reporting of diagnosis by a doctor or other health-care professional. The identification of hypertension was additionally based on measured mean systolic blood pressure/diastolic blood pressure > 140/90 mmHg, or self-reported use of antihypertensive agents, including diuretics, beta-blockers, calcium-channel blockers, angiotensin-converting enzyme (ACE) inhibitors, angiotensin receptor blockers, or other antihypertensive agents (including alpha-1-blockers, central alpha-2-agonists, direct vasodilators, renin inhibitors, and other centrally acting drugs).

To ensure comparability with standard lab assays and consistency when testing methods or instruments change across different survey cycles, NHANES conducts quality assurance and monitoring, and provides specific calibration recommendations in analytical notes for affected lab tests and survey cycles [10]. Serum creatinine levels measured from the 1999–2000, and 2005–2006 survey cycles were calibrated according to NHANES recommended equations to be traceable to an isotope dilution mass spectrometry (IDMS) reference method (calibration not needed for the other survey years in this study). Similarly, urine creatinine measures before the 2007–2008 surveys were corrected according to NHANES recommendations to be consistent with the 2007–2008 survey and forward. Spot urine albumin was measured and tested consistently across 1999–2012 survey cycles. Urinary albumin to creatinine ratio (UACR) was calculated by taking the ratio between urinary albumin and urinary creatinine and expressed as mg/g [14].

CKD was categorized based on the new KDIGO classification recommendations [4]. GFR categories were based on eGFR: G1 ≥ 90 ml/min/1.73 m^2^ (normal to high), G2 60–89 ml/min/1.73 m^2^ (mildly decreased), G3a 45–59 ml/min/1.73 m^2^ (mildly to moderately decreased), G3b 30–44 ml/min/1.73 m^2^ (moderately to severely decreased), G4 15–29 ml/min/1.73 m^2^ (severely decreased), and G5 < 15 ml/min/1.73 m^2^ (kidney failure). Albuminuria categories were based on the UACR: A1 < 30 mg/g (normal to mildly increased), A2 30–300 mg/g (moderately increased), and A3 > 300 mg/g (severely increased). eGFR was calculated using the Chronic Kidney Disease Epidemiology Collaboration (CKD-EPI) equation [14]. Because some practitioners and institutions may still use the Modification of Diet in Renal Disease (MDRD) equation [15], eGFR using this equation was also calculated and reported.

To provide an estimate of the US prevalence of CKD in the overall and age ≥ 65 years T2DM population, the observations from the NHANES were weighted to account for the complex design of NHANES [16] and age-adjusted to the 2012 US diabetes population according to the National Health Interview Survey (NHIS) [17,18]. Statistical analyses were performed using survey procedures in SAS version 9.2 (SAS Institute, Cary, North Carolina, USA). We performed linear trend tests on the projected mean eGFR and UACR values, as well as prevalence of the eGFR and UACR categories by the KDIGO classification. Two-sided p values of < 0.05 were considered statistically significant.

## Results

A total of 2915 adults with T2DM were identified from NHANES 1999–2012. We combined the NHANES 1999–2012 data because none of the linear trend tests for eGFR and UACR were statistically significant, and numerical values also appeared comparable across these survey cycles (data not shown). The sample demographics and projected national estimates are presented in Table 1. Based on the national estimates, the mean standard error (SE) age of the US T2DM population was 61.4 (0.4) years, with 50.3% age ≥ 65 years. The mean (SE) duration of T2DM was 9.7 (0.1) years. Non-Hispanic whites comprised 62.6% of the study population, non-Hispanic blacks 15.9%, and Mexican Americans 8.1%. The most common self-reported comorbidities were hypertension (71.9%), retinopathy (20.9%), coronary heart disease (12.5%), myocardial infarction (12.0%), and congestive heart failure (10.6%).

**Table 1:** Demographic characteristics

**Variable**	**Overall**		**Age ≥ 65 years**	
	**NHANES**	**Projected national estimate**	**NHANES**	**Projected national estimate**
	**N = 2915**		**N = 1466**	
Mean (SE) age, years	63.9 (0.2)	61.4 (0.4)	73.4 (0.1)	73.1 (0.1)
Age ≥ 65 years, % (n)	50.3 (1466)	40.3 (N/A)	100 (1466)	100 (N/A)
Sex, % male (n)	52.0 (1517)	50.8 (N/A)	51.8 (760)	47.1 (N/A)
Mean (SE) diabetes duration, years	10.7 (0.2)	9.7 (0.2)	13.2 (0.3)	12.9 (0.3)
Mean (SE) eGFR (CKD-EPI), ml/min/1.73 m^2^	78.2 (0.5)	80.5 (0.5)	65.3 (0.6)	64.2 (0.8)
Mean (SE) eGFR (MDRD), ml/min/1.73 m^2^	78.5 (0.5)	79.1 (0.6)	66.7 (0.6)	64.8 (0.8)
Mean (SE) UAE, mg/g	109.7 (1.3)	109.1 (1.4)	103.4 (1.6)	99.1 (1.8)
**Race/ethnicity**				
Mexican American, % (n)	21.9 (638)	8.1 (N/A)	18.4 (269)	5.0 (N/A^a^)
Other Hispanic, % (n)	7.9 (231)	5.9 (N/A)	6.0 (88)	3.4 (N/A^a^)
Non-Hispanic white, % (n)	37.9 (1105)	62.6 (N/A)	47.0 (689)	72.6 (N/A^a^)
Non-Hispanic black, % (n)	26.5 (773)	15.9 (N/A)	23.1 (338)	12.4 (N/A^a^)
Other, including multiracial, % (n)	5.8 (168)	7.4 (N/A)	5.6 (82)	6.5 (N/A^a^)
**Comorbidities (self-reported)**				
Hypertension, % (n)^a^	74.0 (2157)	71.9 (N/A)	80.3 (1177)	80.9 (N/A^a^)
Retinopathy, % (n)	22.1 (645)	20.9 (N/A)	23.1 (339)	22.2 (N/A^a^)
Coronary heart disease, % (n)	12.0 (351)	12.5 (N/A)	16.6 (243)	19.9 (N/A^a^)
Myocardial infarction, % (n)	12.7 (371)	12.0 (N/A)	16.5 (241)	18.2 (N/A^a^)
Congestive heart failure, % (n)	11.2 (326)	10.6 (N/A)	15.5 (227)	16.9 (N/A^a^)
Angina, % (n)	9.0 (261)	9.5 (N/A)	11.4 (167)	13.9 (N/A^a^)
Stroke, % (n)	10.1 (293)	9.1 (N/A)	13.6 (200)	14.4 (N/A^a^)

^a^N = 21,318,626 based on the total number of persons with diabetes who were age ≥ 18 years, NHIS 2012.

The national estimate of mean (SE) eGFR (CKD-EPI) for the T2DM population was 80.5 (0.5) ml/min/1.73 m^2^, and the mean (SE) urinary albumin excretion (UAE) was 109.1 (1.4) mg/g. The overall prevalence of CKD based on either eGFR (CKD-EPI) or albuminuria criteria was 43.5%, with 95% confidence interval (CI) 41.6-45.4%. The prevalence of mildly decreased to more severe renal impairment based on eGFR below the threshold of 60 ml/min/1.73 m^2^ was 22% (95% CI 20.4-23.5%). Based on the new subdivision of stage 3 CKD into 3a/3b, the prevalence of Stage 3a CKD (eGFR > 45–59 ml/min/1.73 m^2^) was 12.9% (95% CI 11.5-14.3%) (Table 2) and Stage 3b or lower (eGFR < 45 ml/min/1.73 m^2^) was 9% (95% CI 7.8-10.3%). The prevalence of elevated UAE based on UACR ≥ 30 mg/g was 32.2% (95% CI 30.0-34.3%), 24.4% (95% CI 22.3-26.4%) with moderately increased (UACR ≥ 30–300 mg/g) and 7.8% (95% CI 6.5-9.1%) with severely increased (UACR > 300 mg/g) UAE.

**Table 2:** **eGFR (CKD-EPI) and UACR category prevalence of CKD in T2DM**^**a**^

**GFR category, ml/min/1.73 m**^**2 **^**(CKD-EPI)**	**Albuminuria category, mg/g**							
	**< 30**		**30-300**		**> 300**		**Row**^**c**^	
	**N**	**% (95% CI)**	**N**	**% (95% CI)**	**N**	**% (95% CI)**	**N**	**% (95% CI)**
**G1** ≥ **90**	733^b^	29.1 (27.0-31.2)	269	9.2 (7.8-10.5)	56	1.4 (0.9-1.9)	1058	39.7 (37.4-42.0)
**G2 60-89**	753^b^	27.4 (25.1-29.6)	282	8.4 (7.0-9.7)	92	2.6 (1.9-3.2)	1127	38.3 (35.7-40.9)
**G3a 45-59**	242	8.0 (6.9-9.2)	132	3.6 (2.8-4.3)	49	1.4 (0.8-1.9)	423	12.9 (11.5-14.3)
**G3b 30-44**	84	2.7 (2.1-3.3)	75	2.1 (1.5-2.6)	36	1.0 (0.5-1.5)	195	5.8 (4.8-6.8)
**G4 15-29**	20	0.6 (0.2-0.9)	33	1.2 (0.6-1.7)	36	1.0 (0.5-1.6)	89	2.8 (2.0-3.6)
**G5 < 15**	^d^	0.0 (0.0-0.1)	^d^	0.0 (0.0-0.0)	20	0.4 (0.1-0.6)	23	0.4 (0.2-0.7)
**Column N, % (95% CI)**^**c**^	1834	67.8 (65.7-70.0)	792	24.4 (22.3-26.4)	289	7.8 (6.5-9.1)	2915	100 (N/A)

^a^Age adjusted to 2012 NHIS diabetes population.

^b^Does not meet CKD criteria based on eGFR or albuminuria.

^c^Sum of rows and columns may deviate due to rounding.

^d^Cell frequency suppressed when count < 3 to avoid potential identifiability and imprecision of estimates.

Using MDRD to calculate eGFR, the mean (SE) eGFR was 79.1 (0.6) ml/min/1.73 m^2^. The overall prevalence of CKD using either eGFR (MDRD) or albuminuria criteria was 45.1%, 95% CI 43.0-47.1%. The prevalence of mildly decreased to more severe renal impairment based on eGFR below the threshold of 60 ml/min/1.73 m^2^ was 24.0%, 95% CI 22.1-26.0%; 14.9% (95% CI 13.2-16.6%) had eGFR (MDRD) 45–59 ml/min/1.73 m^2^ (Table 3) and 9.2% had eGFR (MDRD) < 45 ml/min/1.73 m^2^.

**Table 3:** **eGFR (MDRD) and UACR category prevalence of CKD in T2DM**^**a**^

**GFR category, ml/min/1.73 m**^**2 **^**(MDRD)**	**Albuminuria category, mg/g**							
	**< 30**		**30-300**		**> 300**		**Row**^**c**^	
	**N**	**% (95% CI)**	**N**	**% (95% CI)**	**N**	**% (95% CI)**	**N**	**% (95% CI)**
**G1** ≥ **90**	629^b^	22.9 (20.7-25.1)	239	7.4 (6.1-8.6)	46	1.2 (0.7-1.7)	914	31.4 (28.9-34.0)
**G2 60-89**	834^b^	32.1 (29.4-34.7)	305	9.8 (8.3-11.3)	97	2.7 (2.0-3.3)	1236	44.6 (41.7-47.5)
**G3a 45-59**	267	9.6 (8.2-11.0)	141	4.0 (3.0-4.9)	51	1.4 (0.8-1.9)	459	14.9 (13.2-16.6)
**G3b 30-44**	88	2.9 (2.2-3.7)	74	2.1 (1.5-2.6)	40	1.2 (0.7-1.7)	202	6.2 (5.1-7.2)
**G4 15-29**	15	0.4 (0.2-0.5)	32	1.2 (0.6-1.7)	35	1.0 (0.4-1.6)	82	2.5 (1.8-3.2)
**G5 < 15**	^d^	0.0 (0.0-0.0)	^d^	0.0 (0.0-0.0)	20	0.4 (0.1-0.6)	22	0.5 (0.1-0.7)
**Column N, % (95% CI)**^**c**^	1834	67.8 (65.7-70.0)	792	24.4 (22.3-26.4)	289	7.8 (6.5-9.1)	2915	100 (N/A)

^a^Age adjusted to 2012 NHIS diabetes population.

^b^Does not meet CKD criteria based on eGFR or albuminuria.

^c^ Sum of rows and columns may deviate due to rounding.

^d^Cell frequency suppressed when count < 3 to avoid potential identifiability and imprecision of estimates.

A total of 1466 NHANES participants were aged ≥ 65 years, representing 50.3% of the analysis sample. The projected mean (SE) age for this population was 73.4 (0.1) years, and the mean (SE) diabetes duration was 12.9 (0.3) years. Non-Hispanic whites comprised 72.6% of the study population age ≥ 65 years, non-Hispanic blacks 12.4%, and Mexican Americans 5.0%. The most common self-reported comorbidities were hypertension (80.9%), retinopathy (22.2%), coronary heart disease (19.9%), myocardial infarction (18.2%), and congestive heart failure (16.9%).

For the age ≥ 65 years subgroup, the prevalence of CKD using either eGFR (CKD-EPI) or albuminuria criteria was 61.0%, 95% CI (57.9- 64.1%). The prevalence of mildly decreased to more severe renal impairment based on eGFR (CKD-EPI) below the threshold of 60 ml/min/1.73 m^2^ was 43.1% (95% CI 39.5-46.6%). Stage G3a (eGFR 45–59 ml/min/1.73 m^2^) represented 24.5% (95% CI 21.6-27.4%) (Table 4), and eGFR < 45 ml/min/1.73 m^2^ was 18.6% (95% CI 15.9-21.2%). The prevalence of increased UAE based on UACR ≥ 30 mg/g was 39.1% (95% CI 36.3-41.8%). Moderately increased UAE (ACR 30–300 mg/g) was present in 29.3% (95% CI 26.4-32.2%), and severely increased UAE (ACR > 300 mg/g) was present in 9.8% (95% CI 7.5-12.0%).

**Table 4:** **Age ≥ 65 years: eGFR (CKD-EPI) and ACR category prevalence of CKD in T2DM**^**a**^

**GFR category, ml/min/1.73 m**^**2 **^**(CKD-EPI)**	**Albuminuria category, mg/g**							
	**< 30**		**30-300**		**> 300**		**Row**^**c**^	
	**N**	**% (95% CI)**	**N**	**% (95% CI)**	**N**	**% (95% CI)**	**N**	**% (95% CI)**
G1 ≥ 90	120^b^	6.0 (4.7-7.3)	54	3.2 (2.2-4.1)	7	0.2 (0.0-0.3)	181	9.4 (7.9-10.9)
G2 60-89	448^b^	33.0 (30.3-35.8)	187	11.5 (9.5-13.6)	51	3.1 (2.0-4.1)	686	47.6 (44.4-50.8)
G3a 45-59	197	14.8 (12.6-17.0)	111	7.5 (5.9-9.2)	35	2.2 (1.2-3.2)	343	24.5 (21.6-27.4)
G3b 30-44	69	5.6 (4.2%-7.0)	68	4.4 (3.2-5.7)	31	2.1 (1.0-3.3)	168	12.2 (10.0-14.4)
G4 15-29	19	1.4 (0.6-2.2)	28	2.7 (1.4-4.0)	29	2.0 (0.9-3.1)	76	6.1 (4.5-7.8)
G5 < 15	^d^	0.1 (-0.1-0.2)	^d^	0.0 (0.0-0.0)	10	0.2 (0.0-0.4)	12	0.3 (0.1-0.5)
**Column N, % (95% CI)**^**c**^	854	60.9 (58.2-63.7)	449	29.3 (26.4-32.2)	163	9.8 (7.5-12.0)	1466	100 (N/A)

^a^Age adjusted to 2012 NHIS diabetes population.

^b^Does not meet CKD criteria based on eGFR or albuminuria.

^c^Sum of rows and columns may deviate due to rounding.

^d^Cell frequency suppressed when count < 3 to avoid potential identifiability and imprecision of estimates.

Using MDRD to calculate eGFR in the age ≥ 65 years subgroup, the mean (SE) eGFR was 64.8 (0.8) ml/min/1.73 m^2^. The overall prevalence of CKD using either eGFR (MDRD) or albuminuria criteria was 61.8% (95% CI 58.5-65.0%). The prevalence of mildly decreased to more severe renal impairment based on eGFR (MDRD) below the threshold of 60 ml/min/1.73 m^2^ was 44.2% (95% CI 40.3-48.1%); 26.1% had eGFR 45–59 ml/min/1.73 m^2^ (95% CI 22.9-29.3%) (Table 5), and 18.1% (95% CI 15.5-20.8%) had eGFR < 45 ml/min/1.73 m^2^.

**Table 5:** **Age ≥ 65 years: eGFR (MDRD) and UACR category prevalence of CKD in T2DM**^**a**^

**GFR category, ml/min/1.73 m**^**2 **^**(MDRD)**	**Albuminuria category, mg/g**							
	**< 30**		**30-300**		**> 300**		**Row**^**c**^	
	**N**	**% (95% CI)**	**N**	**% (95% CI)**	**N**	**% (95% CI)**	**N**	**% (95% CI)**
G1 ≥ 90	136^b^	6.5 (5.3-7.7)	71	4.3 (3.2-5.4)	11	0.6 (0.1-1.1)	218	11.4 (9.9-12.9)
G2 60-89	427^b^	31.7 (28.7-34.8)	168	10.1 (8.1-12.1)	46	2.5 (1.6-3.5)	641	44.4 (40.8-48.0)
G3a 45-59	205	15.8 (13.4-18.2)	116	7.9 (6.2-9.7)	37	2.4 (1.3-3.4)	358	26.1 (22.9-29.3)
G3b 30-44	72	6.1 (4.5-7.7)	66	4.3 (3.1-5.5)	31	2.1 (1.0-3.3)	169	12.5 (10.3-14.8)
G4 15-29	14	0.8 (0.4-1.3)	27	2.6 (1.3-3.9)	28	2.0 (0.9-3.1)	69	5.4 (3.9-6.9)
G5 < 15	^d^	0.0 (0.0-0.0)	^d^	0.0 (0.0-0.0)	10	0.2 (0.0-0.4)	11	0.2 (0.1-0.4)
**Column N, % (95% CI)**^**c**^	854	60.9 (58.2-63.7)	449	29.3 (26.4-32.2)	163	9.8 (7.5-12.0)	1466	100 (N/A)

^a^Age adjusted to 2012 NHIS diabetes population.

^b^Does not meet CKD criteria based on eGFR or albuminuria.

^c^Sum of rows and columns may deviate due to rounding.

^d^Cell frequency suppressed when count < 3 to avoid potential identifiability and imprecision of estimates.

## Discussion

Based on a nationally representative sample, this report is the first to provide estimates of the prevalence of CKD in the US population with T2DM based on the new KDIGO classification. These new estimates provide insights on the prevalence of the GFR categories G3a and G3b and the albuminuria categories. A recent report by Plantinga *et al.*[19] using NHANES 1999–2006 and a similar staging of CKD reported a slightly lower prevalence of CKD than this study. The Plantinga *et al.* report was based on a smaller sample size (the more recent NHANES data were not available at the time of their study) with a lower mean age, and did not attempt to exclude T1DM or those diagnosed with T2DM age < 30 years. A previous report by Coresh *et al.*[20] observed that the prevalence of CKD increased with age, suggesting that the criteria used for this study may explain some of the differences observed. Additionally, the previous report did not provide the distribution of albuminuria categories as it predated the new KDIGO classification.

Another recent report on the prevalence of CKD in diabetes based on NHANES 2005–2010 observed a lower prevalence of CKD [2]. This analysis included both T1DM and T2DM, and included NHANES participants age ≥ 20 years, which may explain the differences in CKD prevalence observations. Additionally, the analysis did not report details regarding the severity of albuminuria.

Consistent with previous reports, the overall prevalence of CKD based on eGFR was lower using CKD-EPI compared to MDRD [21,22]. Based on the evidence that CKD-EPI is more accurate than MDRD for estimating kidney function and predicting risk of mortality and ESRD (KDIGO), CKD-EPI is now the recommended equation. We chose to report eGFR based on MDRD as well, as this equation may still be in use by some clinicians and institutions [23].

The prevalence of CKD in the age ≥ 65 years population was higher than in the overall T2DM population. This is consistent with previous reports of increasing prevalence of CKD [20] and incidence of ESRD [24] with age. The prevalence of comorbidities such as hypertension, retinopathy, and cardiac disease was higher in the age ≥ 65 years population compared to the overall population.

The recognition of the presence of CKD and classification based on the severity of CKD was first proposed by KDOQI in 2002. The basis of the staging was to provide stage-specific treatment recommendations [4,5] and allow determination of risk of progression to ESRD and mortality. Both diabetes-specific and more general treatment recommendations were given. The importance of the inclusion of the level of albuminuria in making treatment decisions was recently emphasized by the results of a meta-analysis by Lv *et al*. [25]. Consideration of kidney function within the categories 3a and 3b when making diabetes- and non-diabetes-related treatment decisions has recently been emphasized [4,26]. Medication-related considerations include the potential for decreased ability to excrete drugs and/or their metabolites, increased sensitivity to medications (e.g. those bound to albumin), diminished tolerance of side effects, or loss of efficacy. The importance of the level of kidney function within categories 3a and 3b is highlighted in KDIGO for metformin, where a review of its continued use is recommended when a patient transitions from category G3a to G3b [4]. Other management-related considerations at the transition of category 3a to 3b CKD include the frequency of recommended monitoring and evaluation for evidence of metabolic bone disease [4]. This current analysis, which includes information related to the severity of albuminuria and the distribution of CKD within categories 3a and 3b, is informative to health-care providers, payers, and policy makers when making decisions on treatment choices and population-based interventions for patients diagnosed with T2DM to potentially prevent the progression of CKD and to minimize the impact of associated complications.

The information related to the T2DM population aged ≥ 65 years may be relevant to those who provide care and make decisions specifically for this population, such as Medicare Part D pharmacy benefit plans. The relevance of this information in this older adult group is highlighted in the ADA 2014 standards of care [9], where there are specific considerations for AHA drug classes such as metformin, thiazolidinediones, insulin secretagogues (such as sulfonylureas), and insulin.

This study has several limitations. We focused specifically on T2DM, and therefore made an attempt to exclude NHANES participants with T1DM based on previously reported methodologies. The empirical algorithm based on age at diabetes onset and early initiation of insulin therapy may exclude younger-onset T2DM patients and include older-onset T1DM patients. The determination of the presence of kidney disease based on eGFR and albuminuria was made based on a single random sample of the laboratory values, so the chronicity of the observations could not be determined. The cause of CKD could not be determined based on the data collected in NHANES, so we could not categorize CKD according to the cause category of the new KDIGO classification. Due to the cross-sectional nature of NHANES, outcomes such as CKD progression and mortality could not be assessed.

## Conclusions

This study confirms the high prevalence of CKD in T2DM, with 43.5% of individuals having evidence of CKD, and provides insights into prevalence of all CKD categories based on the new KDIGO staging system. The prevalence of more severe impairment of renal function, defined as eGFR < 45 ml/min/1.73 m^2^, impacts < 10% of T2DM patients. This study also provides estimates of the distribution of albuminuria by severity within the categories of eGFR, providing insights into the distribution of risk categories for progression to ESRD and mortality. Additionally, this study provides estimates of the prevalence of comorbidities such as hypertension, retinopathy, and cardiac disease in people with T2DM, based on a nationally representative sample. This information may be useful to clinicians, policy makers, and entities focused on population health management.

## Abbreviations

ACE: Angiotensin-converting enzyme; ADA: American Diabetes Association; CGA: *C*ause of CKD, *G*FR category, and Albuminuria category; CI: Confidence interval; CKD: Chronic kidney disease; CKD-EPI: Chronic Kidney Disease Epidemiology Collaboration; eGFR: Estimated glomerular filtration rate; ESRD: End-stage renal disease; IDMS: Isotope dilution mass spectrometry; KDIGO: Kidney Disease: Improving Global Outcomes; KDOQI: Kidney Disease Outcomes Quality Initiative; MDRD: Modification of Diet in Renal Disease; NHANES: National Health and Nutrition Examination Survey; NHIS: National Health Interview Survey; T1DM: Type 1 diabetes mellitus; T2DM: Type 2 diabetes mellitus; UAE: Urinary albumin excretion; UACR: Urinary albumin to creatinine ratio; US: United States.

## Competing interests

RAB and MFTR are employees of Janssen Scientific Affairs, LLC, Raritan, NJ. YW and VZ are employees of Janssen Research & Development, LLC, Titusville, NJ. All authors are stockholders of Johnson and Johnson.

## Authors’ contributions

RAB conceived the study concept, participated in the design of the study, participated in the interpretation of the data, and led the development of the manuscript. YW conceived the study concept, led the design of the study, helped with the statistical analysis, and helped in the development of the manuscript. VZ participated in the design of the study, performed the statistical analysis, and helped in the development of the manuscript. MFTR participated in the design of the study and the interpretation of the data, and helped in the development of the manuscript. All authors have read and approved the final manuscript and have participated in alignment with the ICMJE standards.
